# Cross-kingdom co-occurrence networks in the plant microbiome: Importance and ecological interpretations

**DOI:** 10.3389/fmicb.2022.953300

**Published:** 2022-07-25

**Authors:** Kiseok Keith Lee, Hyun Kim, Yong-Hwan Lee

**Affiliations:** ^1^Department of Agricultural Biotechnology, Seoul National University, Seoul, South Korea; ^2^Interdisciplinary Program in Agricultural Genomics, Seoul National University, Seoul, South Korea; ^3^Center for Plant Microbiome Research, Seoul National University, Seoul, South Korea; ^4^Plant Immunity Research Center, Seoul National University, Seoul, South Korea; ^5^Research Institute of Agriculture and Life Sciences, Seoul National University, Seoul, South Korea

**Keywords:** cross-kingdom interaction, network, plant microbiome, ecological framework, fungi

## Abstract

Microbial co-occurrence network analysis is being widely used for data exploration in plant microbiome research. Still, challenges lie in how well these microbial networks represent natural microbial communities and how well we can interpret and extract eco-evolutionary insights from the networks. Although many technical solutions have been proposed, in this perspective, we touch on the grave problem of kingdom-level bias in network representation and interpretation. We underscore the eco-evolutionary significance of using cross-kingdom (bacterial-fungal) co-occurrence networks to increase the network’s representability of natural communities. To do so, we demonstrate how ecosystem-level interpretation of plant microbiome evolution changes with and without multi-kingdom analysis. Then, to overcome oversimplified interpretation of the networks stemming from the stereotypical dichotomy between bacteria and fungi, we recommend three avenues for ecological interpretation: (1) understanding dynamics and mechanisms of co-occurrence networks through generalized Lotka-Volterra and consumer-resource models, (2) finding alternative ecological explanations for individual negative and positive fungal-bacterial edges, and (3) connecting cross-kingdom networks to abiotic and biotic (host) environments.

## Introduction

Plants are closely associated with microbes from diverse kingdoms, such as bacteria, archaea, protists, oomycetes, and fungi. Host health and its evolutionary trajectory are impacted by host–microbe interactions and the interactions among microbes from multiple kingdoms (Durán et al., 2018; Zhang et al., 2019; Vemuri et al., 2020; Rao et al., 2021). In the past decade, the bacterial microbiome has been a focus of research, but more and more studies are including the fungal microbiome due to its impact on host health and its bacterial microbiome (van der Heijden et al., 2016; Jiang et al., 2017; Nash et al., 2017; Paterson et al., 2017; Laforest-Lapointe and Arrieta, 2018; Bergelson et al., 2019; Forbes et al., 2019; Kapitan et al., 2019; Galloway-Peña and Kontoyiannis, 2020). In the next decade, we anticipate viruses, protists, and oomycetes to be added to microbial surveys to gain a more holistic insight into the ecology and evolution of the microbial ecosystems. Here, we advocate for the fungal kingdom as a proxy for other non-bacterial microbial kingdoms.

Although co-occurrence network analysis has many unresolved intrinsic and technical limitations (Faust, 2021), it remains to be the only explorative data analysis technique that allows researchers to infer microbial interactions with sequence data, especially when inferring inter-kingdom interactions. However, we lack the perspective on why we are using this cross-kingdom co-occurrence network analysis in the first place. In other words, we vaguely assume and agree that simply adding more kingdoms would be worthwhile. Therefore, in this perspective, we demonstrate the utility of using cross-kingdom networks regarding their evolutionary implication in a rice microbiome domestication dataset. Next, a more important aspect we lack is the perspective on how to ecologically interpret the cross-kingdom networks. The current status of research assumes a simplified “competition vs. cooperation” framework between the bacterial and fungal kingdom, where studies hypothesize that host properties emerge due to either antagonistic or mutualistic relationships between the two kingdoms. Although it is extremely difficult to think beyond the negative/positive edges (correlations) given by the co-occurrence networks, it is paramount to adopt a more multi-dimensional, alternative framework derived from community ecology theories and models. Even if these theories do not provide exclusive mechanistic explanations of the microbial networks, they will still prevent us from designing follow-up experiments that may just increase our confirmation bias and make us experiment with more creative hypotheses to explain the co-occurrence networks. The goal of this perspective is to convey the importance of cross-kingdom networks and provide ecological frameworks to interpret these networks.

## Importance of constructing cross-kingdom networks

By using cross-kingdom networks, researchers revealed that non-bacterial species can act as central hub species in a microbial community and that non-bacterial species can affect community stability and connectivity. We briefly summarize the literature first. Then, to further demonstrate the evolutionary significance of cross-kingdom networks in a plant-microbiome co-evolution context, we use a published rice seed microbiome network dataset.

## Overview of literature using cross-kingdom networks

Cross-kingdom network analysis contributed to the discovery of hub or keystone fungal species in the microbiome (Agler et al., 2016; Banerjee et al., 2016; Tipton et al., 2018; Bergelson et al., 2019; Kim et al., 2020; Lemoinne et al., 2020). Hub species have large network centrality values, such as degree centrality and betweenness centrality, whereas keystone species have a disproportionate destabilizing effect on the community upon their removal. In a co-occurrence network, hub or keystone species can be inferred by identifying species with the highest network centrality indices or using *in silico* extinction methods (Berry and Widder, 2014; Agler et al., 2016). In leaves of *Arabidopsis thaliana*, a fungal species, *Dioszegia* sp. and an oomycetes *Albugo* sp. were detected as hubs in the networks and experimentally validated as keystone species (Agler et al., 2016). In the wild rice seed microbiome, two fungal species were discovered to be hub species in the cross-kingdom network (Kim et al., 2020). Similarly, in the human gut and skin, fungal hubs (e.g., *Davidiellaceae* family, *Candida* spp.) were prevalent (Tipton et al., 2018; Lemoinne et al., 2020). Therefore, bacteria-only research would have neglected these hub/keystone non-bacterial species.

Although little research has focused on the effect of fungal species on the overall connectivity or stability in the plant microbiome, in the human lung and skin microbiomes cross-kingdom networks have higher connectivity and network robustness than single-kingdom networks (Tipton et al., 2018). This suggests that fungi stabilize and facilitate communication in microbial ecosystems (Tipton et al., 2018). The difference between cross-kingdom and bacteria-only networks could indicate the involvement of cross-kingdom interactions in governing microbial community structure. This could lead to the discovery of topological patterns that reflect ecological shifts or co-evolution (Cosetta and Wolfe, 2019; Kim et al., 2020). Accordingly, we use a published network dataset to show that cross-kingdom networks are vital to understand co-evolution in plants.

## Cross-kingdom network analysis with wild and domesticated rice microbiome data

To demonstrate that important aspects of host evolution can be missed when using bacteria-only networks compared to using bacterial-fungal co-occurrence networks, we use a published amplicon sequencing dataset (Kim et al., 2020) of bacterial and fungal endophytic communities in the seeds of 43 rice accessions (17 wild and 26 domesticated accessions). Sixteen accessions of the wild rice were obtained from the International Rice Research Institute (IRRI), Philippines. Grains of 27 rice accessions (one wild and 26 domesticated accessions) were obtained from the National Agrodiversity Center at the National Institute of Agricultural Sciences, Korea. The variation and hierarchical analysis of the seed microbiome data corroborated that seed sampling location had no significant or little effect on the microbial composition compared to the domestication status of the sample (Kim et al., 2020), and thus our discussions will focus on the domestication effect. To help facilitate our discussion, we have explained the terminology related to networks and ecology in the Appendix.

**Figure 1:**
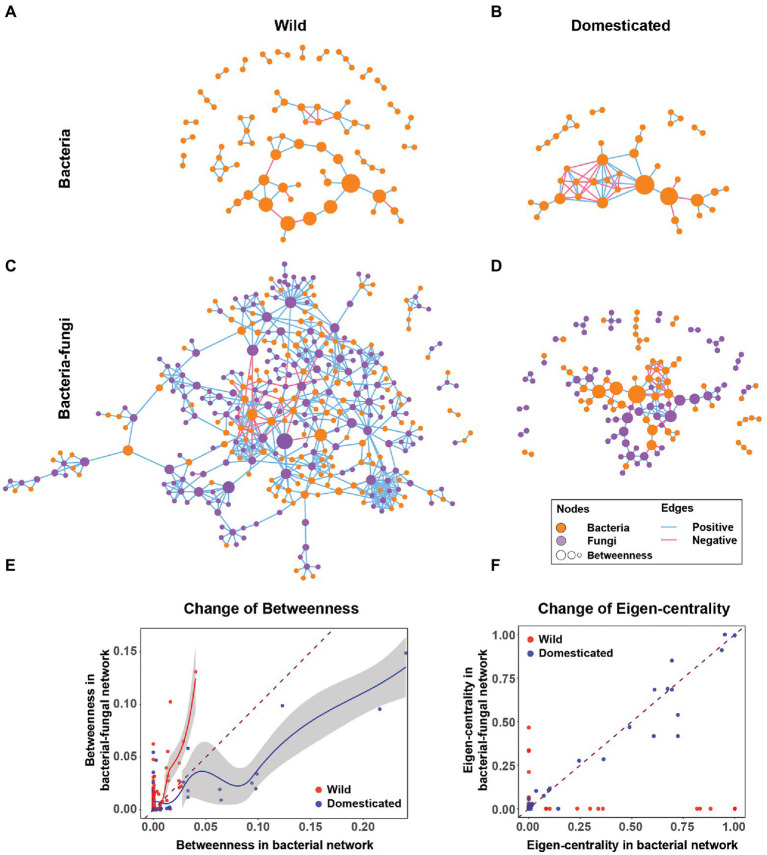
Cross-kingdom networks and bacteria-only networks in wild and domesticated rice seed microbiomes. **(A,B)** Bacteria-only co-occurrence network of wild and domesticated rice seed microbiomes. **(C,D)** Cross-kingdom (bacterial-fungal) co-occurrence network of wild and domesticated rice-seed microbiomes. Orange nodes are bacterial nodes. Purple nodes are fungal nodes. The size of the node is proportionate to betweenness centrality. Blue edges are co-occurrence network edges with positive correlation coefficients. Red edges are co-occurrence network edges with negative correlation coefficients. **(E)** Change of betweenness centrality of the same bacterial nodes in the bacteria-only network (*x*-axis) and bacterial-fungal network (*y*-axis). **(F)** Change in eigenvector centrality of the same bacterial nodes from a bacteria-only network (*x*-axis) to a bacterial-fungal network (*y*-axis). The dashed line is *y* = *x*. Solid lines (red and blue) are regression curves using locally weighted smoothing (loess) to wild and domesticated nodes, respectively. The grey area next to the regression curves indicates a 95% CI.

The microbial co-occurrence network of rice seeds showed that rice domestication alters the rice seed microbiome structure. Including fungi changed the rice seed microbiome network structure in terms of node centrality, network robustness (an aspect of stability), connectance, transitivity, modularity, and nestedness. For the analysis, four co-occurrence networks were created for comparison: a bacterial-fungal network of wild rice, a bacteria-only network of wild rice, a bacterial-fungal network of domesticated rice, and a bacteria-only network of domesticated rice (Figure 1). Network visualizations indicated a stark contrast between bacteria-only and bacterial-fungal networks of wild and domesticated rice microbiomes (Figures 1A–D). For example, the cross-kingdom network of wild rice (Figure 1C) has a 4-fold greater number of nodes (361 vs. 87 nodes) and 8-fold more edges (673 vs. 80 edges) compared to the bacteria-only wild rice network (Figure 1A). In this section, we connect these contrasting network features with host evolution and ecological interactions of bacteria and fungi to demonstrate the importance of cross-kingdom networks.

### Cross-kingdom networks reveal the evolution of the fungal-bacterial relationship during crop domestication

The addition of fungi alters bacterial node centrality in wild and domesticated rice. The addition of fungi boosted bacterial node centrality for most nodes of the wild rice network (Figure 1E). In contrast, in the domesticated rice network, fungi decreased the betweenness centrality of bacteria (Figure 1E). This suggests that domestication selects bacterial species less connected to fungi. Furthermore, an increase in the betweenness centrality of bacteria in the wild rice network suggests that fungal nodes in wild rice act more strongly as modular connectors that create links between bacterial nodes compared to the domesticated rice network. Another important caveat is that fungi shift influential bacterial species in terms of eigenvector centrality in the wild rice network. By contrast, in the domesticated rice networks, the importance of bacterial nodes is unaffected by fungi (Figure 1F). Therefore, cross-kingdom co-occurrence networks reveal that the fungal-bacterial relationship evolved distinctly in wild and domesticated plants.

### Cross-kingdom networks unveil fungal impact on microbiome network stability, transitivity, and modularity

**Figure 2:**
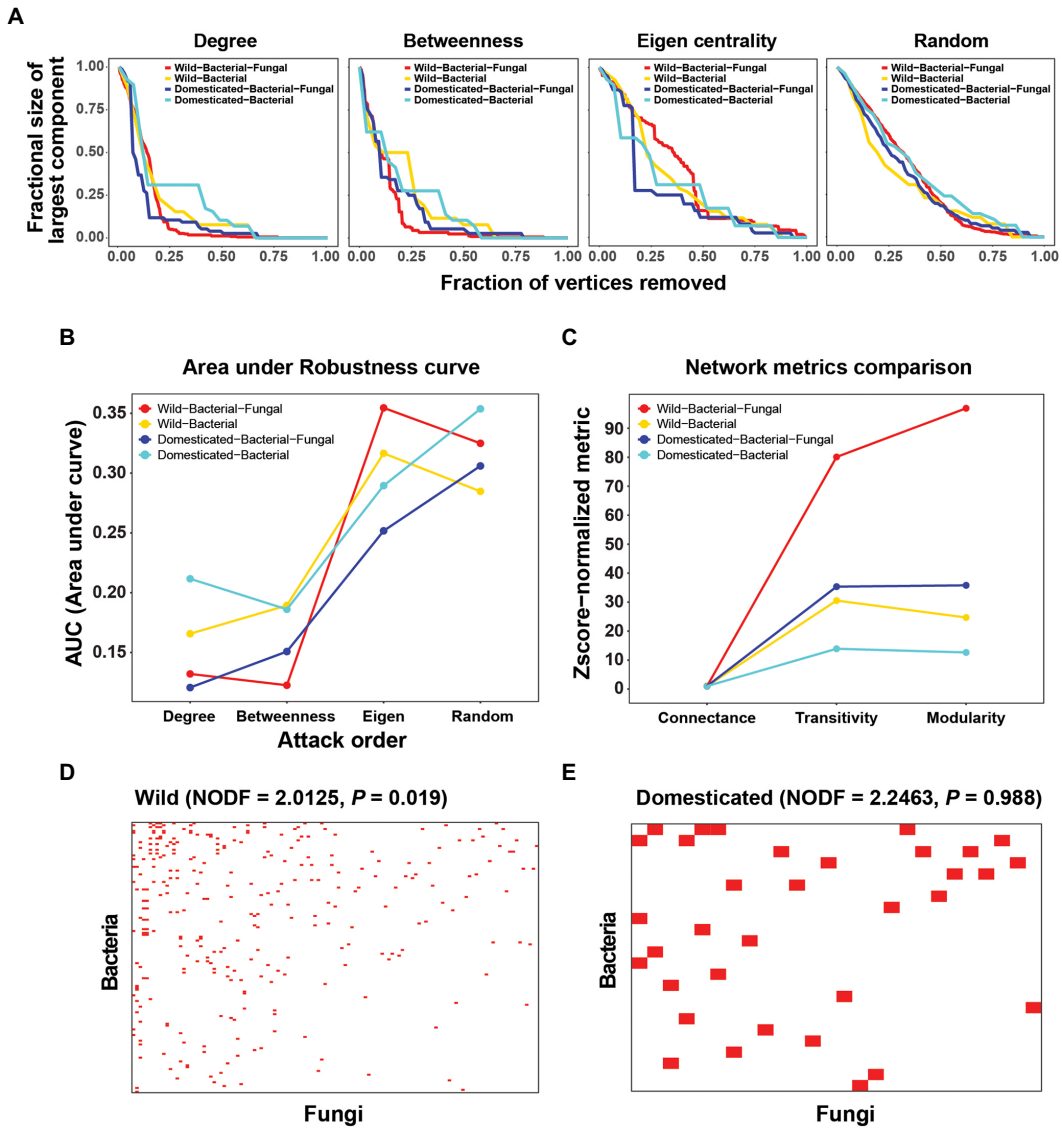
Robustness analysis and connectance, transitivity, modularity, and nestedness of cross-kingdom and bacteria-only networks in wild and domesticated rice seed microbiomes. **(A)** Robustness analysis by *in silico* extinction experiments. Nodes were deleted from the network in order of degree, betweenness centrality, and eigenvector centrality, and randomly. The size of the largest component (*y*-axis) was recorded after every extinction event until all vertices were removed. For robustness curves, the fraction of nodes extinct and the fraction of the largest component size (largest component size after the attack ÷ largest component size before any extinction) were plotted on the *x*- and *y*-axes, respectively. **(B)** Area under the robustness curves for each attack order and network. **(C)** *Z*-score normalized connectance, transitivity, and modularity. *Z*-score normalization of summary statistics was carried out using the mean and SD of random configuration models (with the curveball method). Because the connectance of the random model had a standard deviation of 0, *Z*-score normalization was done by dividing by the mean of the random model. **(D,E)** Visualization of nestedness in wild and domesticated cross-kingdom co-occurrence networks. Rows are bacterial species and columns are fungal species. A red pixel denotes a link between bacterial and fungal species. Only edges connecting bacterial and fungal species were used to create the bipartite network.

Robustness, a measure of network stability, was analyzed *in silico via* extinction experiments. Nodes were deleted from the network in a given order: degree (highest to lowest), betweenness centrality (highest to lowest), eigenvector centrality (highest to lowest), and random deletion (Figure 2A). We considered secondary extinctions as node removal due to the loss of all connected edges. The size of the largest component was recorded after every extinction event. For robustness curves, the fraction of nodes extinct and the fraction of the largest component size (largest component size after attack/largest component size before any extinction) were plotted on the *x* and *y*-axes, respectively.

Connectance, transitivity, modularity, and nestedness metrics were calculated in R or with the Vegan R package. To derive nestedness, only edges connecting bacterial and fungal species were used to create a bipartite network. *Z*-score normalization of summary statistics was carried out using the mean and SD of random configuration models (with the curveball method). Because the connectance of the random model had a standard deviation of 0, the *Z*-score normalization was done by dividing by the mean of the random model.

Network robustness was examined to determine the effect of fungi on the stability of the microbial network. Robustness is a good measure of network stability when time-series data are not available to consider dynamics explicitly. Robustness curves revealed different effects in the attack order (Figure 2A). If the attack order reflects the node’s importance in network stability, deleting nodes in that order will quickly destabilize the network and decrease the size of the largest component. That will make the robustness curves more convex and closer to the plot’s periphery, and thus decrease the area under the robustness curves (AUC). Because random extinctions do not reflect the order of node importance, the robustness curve will be less convex and closer to the diagonal of the plot. We observed that an eigenvector centrality-based attack yielded similar curves to random extinctions. This suggests those degree and betweenness centrality are more reliable measures of node importance in terms of maximum damage from disconnecting the integrity of the system and breaking it into small components. The lower AUC of degree and betweenness centrality attack orders in the bacterial-fungal networks showed that consideration of fungi decreased the network robustness of wild and domesticated rice microbial networks (Figure 2B). These results may hold the key to explaining the mechanism by which fungi reduce diversity and abundance in domesticated rice. We hypothesize that because fungi reduce network stability, selection pressure during domestication may have destroyed bacterial-fungal modules or interactions in the perturbation-susceptible wild rice microbial community.

Moreover, the role of fungi can be evaluated using network summary statistics. For wild and domesticated rice networks, fungi increased the *Z*-score normalized transitivity (clustering coefficient) and modularity (Figure 2C). This means that fungi increased the probability of the network having adjacent interconnecting nodes and enhanced the modular structure of the microbial community. The nestedness of the wild rice network was significant compared to randomized networks (NODF = 2.0125, *p* = 0.019), but that of the domesticated rice network was not (*p* = 0.988; Figures 2D,E). Network nestedness measures the tendency for nodes to interact with subsets of the interaction partners of better-connected nodes. Therefore, nestedness found only in wild rice indicates that the wild rice microbiome preserves a structured gradient of fungal-bacterial interactions, whereas the domesticated microbiome has randomized non-structured cross-kingdom interactions, possibly disrupted during host domestication.

The use of only bacterial sequence data would have generated a completely different picture of microbe-plant co-evolution. Fungi considerably impacted bacterial centrality by acting as modular connectors and generalist interactors and shifted the order of bacterial influence as measured by eigenvector centrality. Fungi also increased the sensitivity to extinction events in the networks. More importantly, the effect of crop domestication on the microbial community was more evident using bacterial-fungal networks compared to bacterial networks.

## Ecological interpretations of cross-kingdom networks

**Figure 3:**
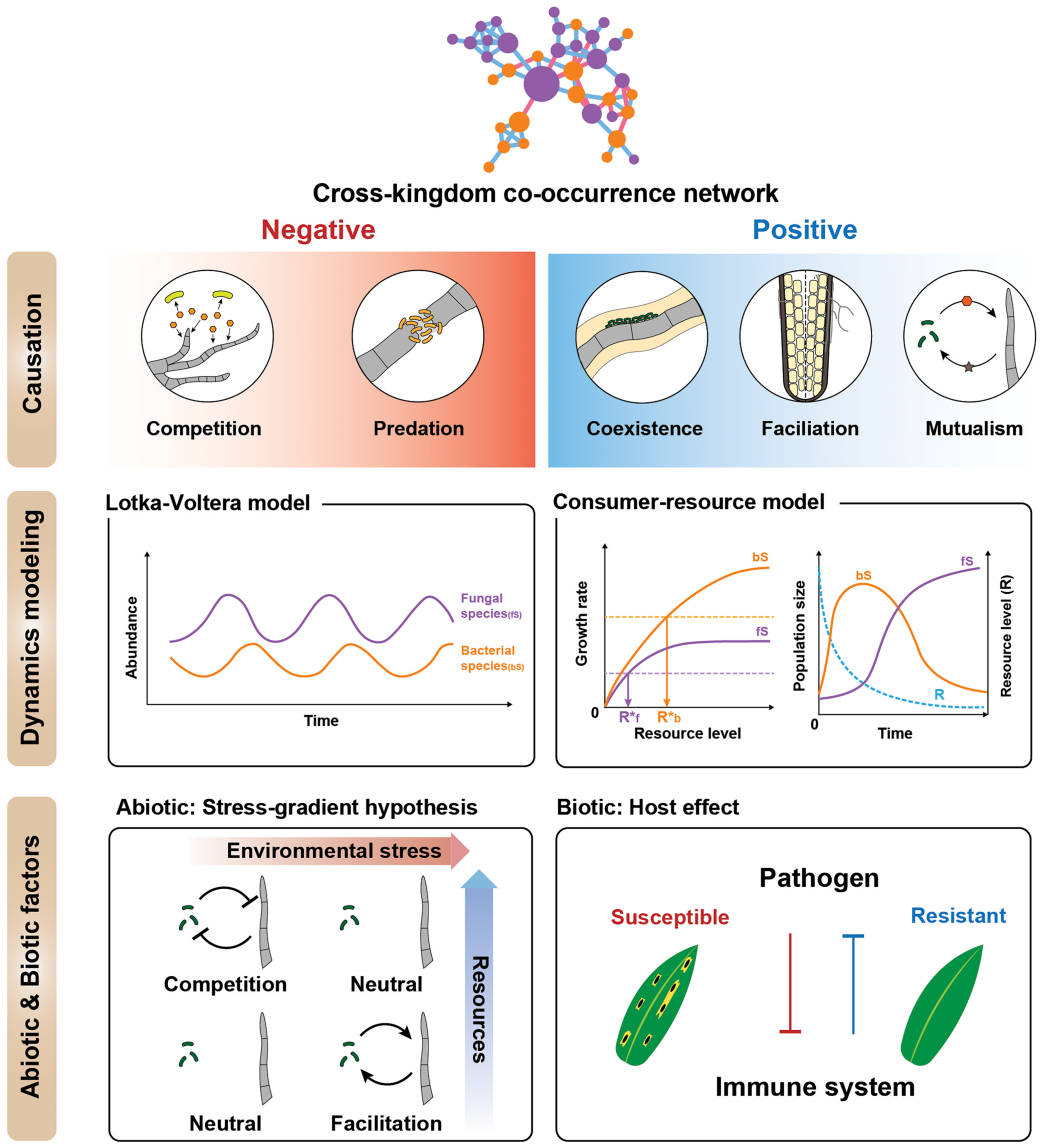
Ecological interpretations of cross-kingdom co-occurrence networks. (Top) Cartoon illustrating ways to interpret individual negative and positive fungal-bacterial edges. Negative interactions include competition and predator–prey relationships, whereas positive interactions are cooperative, such as coexistence, facilitation, and mutualism. All positive and negative correlations are not cooperation and competition, respectively. (Middle) Dynamical modeling of co-occurrence networks using generalized Lotka-Volterra (gLV) and consumer-resource models. The cartoon depicts species dynamics related to the gLV and consumer-resource model, respectively (only for illustration purposes). These models can be used to supplement the network to elucidate the mechanism of the interactions. (Bottom) Effects of abiotic and biotic factors on cross-kingdom networks. To investigate cross-kingdom interactions in natural microbial communities, it is important to treat microbial interactions as variables dependent on the magnitude of stress, space, time (abiotic factors), and host (biotic factor). **(Left)** Stress gradient hypothesis—the relative importance of competitive vs. facilitative interactions varies along the environmental harshness gradient. Another consideration is the host effect (biotic factor). **(Right)** The host immune system mediates the interaction between the bacterial and fungal communities. The biotic factors influencing the microbial community can be affected by host plant pathogen susceptibility or resistance.

The potential of cross-kingdom networks does not end with network centrality or stability analysis. In combination with community ecology models and theories, cross-kingdom networks can generate testable hypotheses. Community ecology aims to understand the temporal and spatial dynamics of communities, interactions of the members, and the emergent properties of communities (Christian et al., 2015). Here, we use community ecology frameworks to suggest how cross-kingdom networks can be ecologically explained and experimentally validated (Figure 3).

### Understanding dynamics and mechanism of co-occurrence networks through generalized Lotka-Volterra and consumer-resource models

A co-occurrence network is a cross-sectional summary of pair-wise microbial interactions based on correlations. Therefore, to further understand ecosystem dynamics and its causal mechanism, the network can be complemented with community population models (e.g., Lotka-Volterra, consumer-resource models). Microbial community population dynamics can be modeled using the generalized Lotka-Volterra (gLV) model in the form:


$$\frac{dx\left(t\right)}{dt}=D\left(x\left(t\right)\right)\;\left(r+Ax\left(t\right)\right)$$

where *x*(*t*) is a species abundance vector of length *n* containing the abundances of all species at time *t*, *r* is a vector of intrinsic growth rates in monoculture, and *A* is an *n*-by-*n* matrix of species-to-species interaction coefficients, thereby called community matrix. For example, *A*_ij_ > 0 denotes that species *i* increases the abundance of species *j*, and *A*_ji_ > 0 denotes species *j* increases species *i*’s abundance. *D*(*x*(*t*)) is a diagonal matrix with species abundance vector *x* on the diagonal. By constructing a gLV model, we can phenomenologically summarize the underlying ecology: biological growth (*r*) and species interaction type and strength (*A*). To infer these gLV parameters, regularized regression can be done with time-series sequence data with well-developed methods such as LIMIT (Fisher and Mehta, 2014) and MDSINE (Bucci et al., 2016).

If we can fit gLV model parameters with time-series microbiome sequencing, we will be able to simulate and construct co-occurrence networks from the gLV model (Berry and Widder, 2014; Hirano and Takemoto, 2019). This will enable us to explain the ecology behind the observed co-occurrence network. Berry and Widder (2014) devised a method to simulate networks from gLVs by numerically integrating a gLV ordinary differential equation that includes a random subset of species until their abundance reaches a steady state. With many of these recorded relative abundances of steady-state profiles, one can compute the pair-wise correlation of species abundance, which then is visualized in a co-occurrence network. We will be able to quantify how much our model can explain the observed network by comparing the simulated co-occurrence network to the observed co-occurrence network. With the community matrix A of the gLV at hand, we can hypothesize how species interactions resulted in the observed phenomenon (Stein et al., 2013).

To experimentally infer the gLV model, it is imperative to have time-series sequence data. This has been successfully carried out with densely sampled (e.g., 1 day interval) human stool time-series data (Stein et al., 2013; Faust et al., 2018). To our knowledge, gLV modeling has not been done using both bacterial and fungal time-series data. Because of the lowering cost of sequencing, it is becoming ever more feasible to use time-series data to ecologically explain the observed cross-kingdom interactions. The number and interval of time-series sampling and sequencing would depend on how fast the community reaches the stable state, the interval of cyclic fluctuations of abundance, if any, and the frequency of external perturbations. The sampling scheme should sufficiently capture the temporal changes in abundance in order to fit the model.

While the gLV model is phenomenological in explaining species interaction, the consumer-resource model can explain explicitly how resource densities influence consumer population growth rates and how in turn competing consumers affect their resources (Tilman, 1982; Mittelbach and McGill, 2019). A resource is defined as either an abiotic (e.g., minerals, nutrients, and light) or biotic (e.g., prey) factor that positively contributes to the growth rate of the species population and is made unavailable to other species when consumed (Mittelbach and McGill, 2019). Most bacterial-fungal interaction studies focus on interference competition involving microbial compounds, such as antimicrobial peptides (e.g., copsin), biosurfactants (e.g., surfactin and nunamycin), phenol, and quinone derivatives (e.g., penicillin), phenazines, and volatile organic compounds (Li et al., 2020). This means that there is a huge opportunity and gap in knowledge in resource competition between bacteria and fungi. Growth experiments have been done with bacterial and fungal species to test if there is niche overlap in carbon sources (Rousk and Frey, 2015). Some studies also showed that nutrients can promote antagonistic relationships between bacteria and fungi (Bosmans et al., 2016; Zheng et al., 2018). However, no study has used the consumer-resource model to quantitatively show how fungal and bacterial communities interact through resource competition. This framework is powerful, like the gLV model, because it enables the prediction of community dynamics and steady-state community structure, and thus it can mechanistically explain the observed co-occurrence networks through resource competition.

To experimentally fit the consumer-resource model, time-series sequence (species abundance) data plus resource concentration (metabolite flux) data need to be collected. Recently, metabolite and species abundance dynamics were successfully predicted using a consumer-resource model in bacterial communities (Gowda et al., 2022). With a small size cross-kingdom community and a focused pool of metabolites, it will be possible to learn the resource-mediated cross-kingdom interactions and its ramification on the co-occurrence network. Together, the gLV and consumer-resource models can provide mechanistic insights and promote the investigation of complex cross-kingdom relationships in networks.

### Interpreting negative and positive fungal-bacterial interactions in cross-kingdom networks

In co-occurrence networks, an edge or a link means that two species’ abundance correlation is significantly negative or positive. Multiple studies have evaluated whether bacterial-fungal interactions are negative or positive in cross-kingdom networks. The results are mixed. For example, bacterial-fungal interactions were found to be mostly antagonistic in the leaf and root of *A. thaliana* (Agler et al., 2016; Durán et al., 2018). Other network studies found that fungal-bacterial edges were dominated by positive links (Bergelson et al., 2019; Kim et al., 2020). In the human skin and lung microbiome, fungal-bacterial edges contributed to an increase in the ratio of negative edges when compared to single-domain networks (Tipton et al., 2018). These mixed results indicate that fungal-bacterial interactions are context-dependent and thus cannot be simply generalized to be either positive or negative. Therefore, an ecological understanding of these positive/negative interactions is needed to understand the nature of inter-kingdom interaction and understand how it affects the community-level structure.

When discussing negative or positive edges in cross-kingdom networks, it is tempting to consider the two microbial kingdoms as simply competitive or cooperative (Agler et al., 2016; Durán et al., 2018). Because this is using correlation as a proxy for causality, one should be careful in presuming that a negative association indicates a competitive relationship or a positive association a cooperative relationship between bacteria and fungi. Here, with the help of community ecology, we describe the possible alternative interpretations of negative and positive correlations of individual fungi and bacteria and avenues to test these hypotheses.

### Predator-prey interactions are underestimated in cross-kingdom networks

A negative correlation does not always imply competition; predation, often overlooked, is another possibility. Predator–prey interactions between bacterial and fungal species have been investigated (Pion et al., 2013; Rudnick et al., 2015; Ballhausen and de Boer, 2016; Swain et al., 2017). For example, the fungus *Morchella* reportedly rears and consumes *Pseudomonas putida* (Pion et al., 2013). Conversely, bacterial mycophagy has been found (Höppener-Ogawa et al., 2009; Ballhausen et al., 2015). Whether the predator is a generalist or a specialist determines the strength of the negative interactions. Generalist predators consume a range of prey species, whereas the feeding choice of specialist predators is restricted to a single species. Thus, specialist fungal/bacterial predation relationships have stronger negative correlations than generalists. Although studies have shown the presence of individual cases of predator–prey relationship between bacteria and fungi, not much empirical research has been done to show how these relationships impact the overall community structure.

Discriminating competition and predation is important because of their different effects on microbiome properties such as stability and invasiveness (Case, 1990; May, 2019). Microbial communities are thought to have a stable equilibrium to which the community returns after perturbations, stochastic events, and temporal changes (Beisner et al., 2003; Lozupone et al., 2012). Simulations indicate that competitive and mutualistic interactions destabilize, whereas predator–prey interactions stabilize the community (Allesina and Tang, 2012). For example, generalist predators (e.g., protists) coexist with prey species in multitrophic systems and stabilize the community by resource partitioning between predators and ensuring the availability of resources for prey (e.g., bacteria; Johnke et al., 2017).

Despite its importance, the predator–prey relationship has been scarcely studied in cross-kingdom co-occurrence networks due to a need for additional validation. With only the co-occurrence network, it is hard to discern predation from competition, because constructed co-occurrence networks are mostly undirected, having no direction in their edges. To circumvent this limitation, one computational strategy is to use time-series abundance data as described in the previous section to compute the community matrix A in the gLV model. When *A*_ij_ < 0 and *A*_ji_ > 0, we can say that species *i* preys on species *j*. Conversely, if *A*_ij_ < 0 and *A*_ji_ < 0, we can assume that species *i* and *j* are in a competitive relationship. However, a simulation study reported that interaction patterns in predator–prey communities were relatively harder to predict compared to those in competitive communities (Hirano and Takemoto, 2019). Therefore, we can supplement sequencing-based methods with experimental methods such as microfluidics, microscopy, and stable isotope probing (Mandolini et al., 2021). Microfluidics will help emulate the natural plant microbiome environment but simplify it so that we can observe predator–prey behavior, and thus it will enable relatively high-throughput screening of predation. In a recreated environment or natural environment, we can use microscopy accompanied by taxa-specific staining (FISH) to track the physical interactions of bacterial and fungal strains. Because microscopic methods might not be sufficient to verify one species consuming another, ^13^C-labeled bacteria or fungi can be deployed to detect whether the predator consumed the isotope-labeled species in microcosm experiments.

### Positive edges may not be cooperation but alleviated competition: Co-existence

Positive associations between bacteria and fungi are commonly regarded as cooperative interactions such as mutualism and facilitation. Facilitation is defined as an interaction in which the presence of one species alters the environment in a way that enhances the growth, survival, or reproduction of a second, neighboring species (Bronstein, 2009). These facilitation mechanisms are abundant in bacterial-fungal interactions (BFIs). Fungi serve as foundation species in the microbiome by providing structural networks for bacterial transport (i.e., the *fungal highway*; Kohlmeier et al., 2005). Fungal mycelia facilitate bacterial movement to nutrient reservoirs previously unreachable (Worrich et al., 2016) or provide access to fungal metabolites (Stopnisek et al., 2016). Bacteria can also benefit fungal communities; bacterial antibiotic treatments significantly impaired fungal growth and secondary metabolite production (Vahdatzadeh et al., 2015; Mondo et al., 2017; Schulz-Bohm et al., 2017; Uehling et al., 2017).

Mutualism, arguably a subset of facilitation, is a reciprocally positive interaction between species (Mittelbach and McGill, 2019). The mutualism of bacteria and fungi can be too specific to be detected in co-occurrence networks but is pervasive (Lastovetsky et al., 2016; Salvioli et al., 2016; Vannini et al., 2016; Li et al., 2017; Jung et al., 2018). The endosymbiont bacterium *Paraburkholderia rhizoxinica*, which inhabits fungal cytoplasm, provides its host *Rhizopus microsporus* with a toxin that confers pathogenicity on rice (Partida-Martinez and Hertweck, 2005). Without the vertically transferred endobacteria, the fungus stops asexual sporulation and significantly decreases mating (Mondo et al., 2017). The seed-borne plant pathogenic bacterium *Burkholderia glumae* and the plant pathogenic fungus *Fusarium graminearum* interact to promote bacterial survival, bacterial and fungal dispersal, and disease severity in rice plants, despite the production of antifungal toxoflavin by the bacteria (Jung et al., 2018).

Positive edges between fungi and bacteria may lead to the notion that bacterial-fungal interactions are cooperative. Facilitation, mutualism, and symbiosis are indeed contributors to positive correlations, but that does not rule out the competition. Bacteria and fungi can show positive correlations while competing under conditions of spatial, temporal heterogeneity, and constant dispersal (Mittelbach and McGill, 2019).

To achieve stable coexistence and positive correlations, niche separation in space or time may be required. Fungal mycelial structures provide unique niches for bacterial colonization: i.e., the *mycosphere* (Frey-Klett et al., 2011). In soil or plant roots, bacteria colonize the mycospheres of diverse basidiomycetous fungi (Warmink et al., 2009). Fungal mycelia add spatial variation to the microbiome, thus promoting species coexistence. Temporal heterogeneity, which may be caused by fluctuations of nutrient type and level in the human gut or plant rhizosphere, also enables species coexistence, as in nectar yeasts (Letten et al., 2018). The other coexistence scenario is dispersal. The constant supply of species by dispersal could exceed the rate of competitive exclusion. This source-sink coexistence is particularly plausible because of the specialized dispersal structures of fungi (i.e., spores) and bacterial aerosols. For example, fungal endophytes colonizing plants are transmitted horizontally among hosts and vertically *via* seed infections (Rodriguez et al., 2009). The ease of microbial dispersal facilitates stable coexistence.

To validate the mechanism behind the positive relationship between bacterial and fungal species in networks, pair-wise interaction can be examined again with the help of microcosm experiments, microfluidics, microscopy, and stable isotope probing (Mandolini et al., 2021). With the microfluidic spatial design, fungal hyphae were observed to facilitate bacterial dispersal (Mafla-Endara et al., 2021). If the mechanism is resource mediated, microcosm experiments with known resources and communities can help quantitatively understand co-existence using the consumer-resource model. To study the effect of spatial and temporal heterogeneity on bacterial-fungal co-existence, one can examine co-existence profiles and networks under varying degrees of spatial/temporal heterogeneity (Gravuer et al., 2020; Shi et al., 2021), and then compare the result to the bacteria-fungi co-culture without spatial/temporal heterogeneity. If the bacteria and fungi cannot co-exist in a homogeneous environment but can co-exist in heterogeneous conditions, this will indicate coexistence through niche separation of time or space.

### Positive edges require stable cooperation: Black queen hypothesis

To explain the evolution of cooperation by Darwinian natural selection, unless the problem of cheaters is unresolved, the Black Queen hypothesis maintains that microbes shed necessary functions based on self-interest and depend on other organisms for those functions (Morris et al., 2012). Bacterial and fungal functions are often leaky, meaning that they produce resources that benefit others, such as the detoxifying activity of catalase-peroxidase and iron chelators to solubilize iron. Natural selection disfavors communities with large cheater populations because community-level efficiency is decreased when secretion genotypes are concentrated in a few microbes. For natural selection to favor cooperating secretors, there must be a moderate level of genotype mixing of beneficial traits (Oliveira et al., 2014). Many secretion systems are related to the horizontal gene transfer in bacteria (Nogueira et al., 2009; Rankin et al., 2011). Also, inter-kingdom horizontal gene transfer occurs frequently in the mycosphere (Bruto et al., 2014; Nazir et al., 2017; Li et al., 2018). Furthermore, the positive effects of mutualism must be limited to avoid destabilizing the microbial community because mutualism could create a continuous positive feedback loop (Allesina and Tang, 2012). Cooperative interactions could be stabilized by negative density dependence or mutualistic effects that decrease with age or growth stage (Mittelbach and McGill, 2019). Therefore, positive correlations in cross-kingdom inference networks could be explained by the creation of stable cooperation, when there are beneficial leaky traits, horizontal gene transfer of those traits, and moderation of positive feedback loops. To experimentally validate these hypotheses, we can utilize stable isotope probing for tracking leaky compounds and use a genomics approach to detect horizontal gene transfer by constructing phylogenetic trees with horizontal gene transfer candidate genes (Jaramillo et al., 2015).

## Future directions: Incorporating abiotic and biotic environmental factors into cross-kingdom networks

We have discussed how to interpret and validate cross-kingdom networks at the community and individual species-to-species level. Ultimately, it is imperative to contextualize these network interpretations within different environmental factors, because cross-kingdom microbial interactions are sensitive to (a) resource availability, (b) environmental stress, and (c) the host (Chen et al., 2019; Ritter et al., 2021). Regarding resource and environmental stress, the gradient of environmental harshness can influence the relative importance of negative and positive interactions among species (i.e., the stress gradient hypothesis; Mittelbach and McGill, 2019). In benign environments, competitive interactions may dominate, whereas, in stressful environments, facilitative interactions may become more important. For example, increased organic input was correlated with a greater rate of negative interactions between bacteria and fungi (Zheng et al., 2018). Investigating the effects on cross-kingdom ecosystems of resource, environmental stress, space, and time is essential to understanding the responses to environmental factors of complex systems.

Additionally, the host must be incorporated into cross-kingdom microbiome research. The change in host phenotype can systematically alter the cross-kingdom microbial network, indirectly mediating fungal-bacterial interactions. For example, plant immune response can alter rhizosphere community structure (Dudenhöffer et al., 2016; Berendsen et al., 2018) where the bacteria or fungi induce systemic acquired resistance in plants, in turn causing changes in the fungal/bacterial community (Carrión et al., 2019; Seybold et al., 2020). In a study, a plant bacterial endophyte consortium induced local or systemic resistance to fungal pathogens in the roots (Carrión et al., 2019). By contrast, the fungal pathogen *Zymoseptoria tritici* indirectly altered bacterial species abundance by suppressing immune-related plant metabolism, making wheat vulnerable to further infection (Seybold et al., 2020). Therefore, the host effect on bacterial-fungal interactions should also be incorporated as a cause of shifts in fungal-bacterial interactions in cross-kingdom co-occurrence networks.

## Conclusion

In systems where microbes from diverse kingdoms affect the host, investigation of only bacteria might miss important aspects of the microbiome. The low cost of sequencing and the availability of computational tools enable the construction of cross-kingdom co-occurrence networks. Therefore, researchers can include multiple microbial kingdoms at a marginal cost to discover the effect of multi-kingdom interactions on the network during environmental change, including host evolution. To exploit the full potential of the cross-kingdom co-occurrence network, it can be complemented by community ecology models and theories. For example, gLV and consumer-resource models complement cross-kingdom networks by providing a dynamic, mechanistic summary of cross-kingdom interactions at the community level. Furthermore, ecological theories make us rethink negative and positive edges, mostly thought of as competition or cooperation. For instance, negative links in networks can be ascribed to predation as well as competition. Positive links can result from stable coexistence due to spatiotemporal heterogeneity and source-sink dynamics. Finally, these ecological interpretations can change due to abiotic/biotic environments, and thus should be always accompanied by explanations of resources, stress gradients, and the host. Ultimately, we hope this perspective will promote the use of cross-kingdom co-occurrence networks by helping researchers interpret the ecological implications of plant microbiome cross-kingdom co-occurrence networks.

## Author contributions

KL and Y-HL conceived and planned the review. KL performed public data analyses and wrote the original draft. KL and HK contributed to the preparation of the manuscript. KL, HK, and Y-HL contributed to manuscript editing and finalization. All authors contributed to the article and approved the submitted version.

## Funding

This work was supported by the National Research Foundation of Korea (NRF) grants funded by the Ministry of Science and ICT (MSIT; 2020R1A2B5B03096402, 2018R1A5 A1023599, and 2021M3H9A1096935).

## Acknowledgments

KL and HK are grateful for a graduate fellowship from the Brain Korea 21 Plus Program.

## Conflict of interest

The authors declare that the research was conducted in the absence of any commercial or financial relationships that could be construed as a potential conflict of interest.

## Publisher’s note

All claims expressed in this article are solely those of the authors and do not necessarily represent those of their affiliated organizations, or those of the publisher, the editors and the reviewers. Any product that may be evaluated in this article, or claim that may be made by its manufacturer, is not guaranteed or endorsed by the publisher.

## Appendix

**Plant microbiome:** The microbiome is the microbial consortium associated with a host or a defined non-host-related environment. The plant microbiome is the microbial community associated with host plant health, fitness, ecology, or evolution. It could reside within plant tissue (endophyte), on the surface of plant tissue (epiphyte), or close to the root system (rhizosphere).

**Plant domestication:** In the last 12,000 years, humans have used various strategies to artificially select and breed plant species or varieties that suit their needs, such as larger harvest or seed/fruit size. This transition to crops from their wild counterparts is plant domestication.

**Biotic and abiotic factors:** The ecosystem can be categorized as biotic or abiotic factors. Biotic factors are living organisms and abiotic factors are non-living components, such as water, air, or space.

**Co-occurrence network:** A co-occurrence network is a graphical visualization of species correlations and consists of links and nodes. The raw data table lists abundance values with the rows being samples and columns being species. After calculating the correlation of species abundance across samples, cutoff correlation coefficients and *p*-values for generating links are determined. In microbial community co-occurrence networks, the nodes are taxa, and the links indicate (significant) correlations of the abundances of two taxa. Links can have positive or negative correlation coefficients. Here, links typically do not have a direction, resulting in an undirected network.

**Cross-kingdom network:** A cross-kingdom network is a cross-kingdom co-occurrence network. The cross-kingdom co-occurrence network is a co-occurrence network using abundance data on multiple microbial kingdoms.

**Edges, Vertices:** Edges are the links of a network and vertices are its nodes.

**Network centrality:** Network centrality is a measure of node importance considering the network structure. Network centrality includes degree centrality, betweenness centrality, closeness centrality, and eigenvector centrality.

**Hub species:** Hub species are represented by nodes in the network with the largest network centrality values such as degree centrality, betweenness centrality, and eigenvector centrality.

**Keystone species:** Keystone species are nodes with a disproportionate destabilizing effect on the community upon their removal. Keystone species and hub species can overlap, but not all keystone species are hubs (Agler et al., 2016).

**Degree:** Degree centrality is the simplest measure of network centrality. The importance of a node is measured by the number of edges connected thereto.

**Betweenness centrality:** Betweenness centrality is the proportion of cases in which a node lies on the shortest path between all pairs of other nodes. Thus, betweenness centrality measures node importance in terms of the extent to which a node lies on paths between other nodes.

**Eigenvector centrality:** Eigenvector centrality or eigenvector’s centrality measures node importance as a function of the importance of its neighboring nodes. This is because not all neighboring nodes are necessarily equivalent. For example, a node with 100 less important neighbors would have lower eigenvector centrality than a node with 100 more important neighbors, despite their degree centrality being identical.

**Robustness:** Network robustness measures the ability of the network to maintain functionality following sequential node removal/attack. Here, we measured functionality as the size (the number of nodes) of the largest component.

**Largest component:** A component is a subset of nodes in which there is at least one path from each member of that subset to each other member. The largest component is the component with the largest number of nodes.

**Connectance:** Network connectance is the fraction of all possible links that are realized in a network (Dunne et al., 2002).

**Modularity:** Network modularity is the number of edges falling within groups minus the expected number in an equivalent network with edges placed at random. Modularity can be positive or negative, with positive values indicating the presence of community structure. Therefore, community structure can be evaluated by assessing the partitions of a network with large positive modularity values (Newman, 2006).

**Transitivity:** Network transitivity is the probability of the network having adjacent interconnected nodes. Network transitivity can identify tightly connected communities (or clusters, subgroups, cliques).

**Nestedness:** Network nestedness measures the tendency for nodes to interact with subsets of the interaction partners of better-connected nodes (Mariani et al., 2019).

**Niche:** An ecological niche is the range of conditions necessary for the persistence of a species, and includes resources, predators, time, and space.
